# Biodiesel Production Using Lithium Metasilicate Synthesized from Non-Conventional Sources

**DOI:** 10.3390/ma15196753

**Published:** 2022-09-29

**Authors:** Eduardo Coutino-Gonzalez, Mario Ávila-Gutiérrez, Arnold Hernández-Palomares, Lilian I. Olvera, Francisco J. Rodríguez-Valadez, Fabricio Espejel-Ayala

**Affiliations:** 1 Centro de Investigaciones en Óptica, A. C. Lomas del Bosque 115, Colonia Lomas del Campestre, León, Guanajuato 37150, Mexico; 2 Centro de Investigación y Desarrollo Tecnológico en Electroquímica, Parque Tecnológico Querétaro, s/n, Pedro Escobedo, Querétaro 76703, Mexico; 3 Instituto de Investigaciones en Materiales, Universidad Nacional Autónoma de Mexico, Apartado Postal 70-360, CU, Coyoacán, Ciudad de México 04510, Mexico

**Keywords:** biodiesel, catalysis, heterogeneous catalysis, Li_2_SiO_3_, renewable fuels, valorization

## Abstract

A facile and versatile process to produce lithium metasilicate (Li_2_SiO_3_) from non-conventional silicon sources (two different sand sources from the central area of México) was developed. The synthesis protocol based on a solid-state reaction followed by a hydrothermal treatment resulted in highly pure lithium metasilicate, as corroborated by XRD, SEM-EDS, and XPS analysis. Furthermore, lithium metasilicate was used as a heterogeneous catalyst for biodiesel production from soybean oil, where conversion yields were compared according to the silicon source used (based on chemical purity, stability, and yield efficiency). The best performing metasilicate material displayed a maximum of 95.5% of biodiesel conversion under the following conditions: 180 min, 60 °C, 5% catalyst (wt./wt., catalyst-to-oil), and 18:1 (methanol:oil). This contribution opens up alternatives for the production of lithium metasilicate using non-conventional precursors and its use as an alternative catalyst in biodiesel production, displaying better chemical stability against humidity than conventional heterogeneous catalysts.

## 1. Introduction 

A strategy to mitigate the effects produced by greenhouse gases is the use of renewable fuels, for instance, biofuels obtained from domestic (animal, and vegetal oils) and agro-industrial (edible and no-edible oils) organic wastes have gained great attention over the past decades due to their ecological added value and low production costs [1]. It has been pointed by several authors that a production chain where biofuels are employed could favor the construction of a circular economy [2,3,4,5]. However, it is necessary to continuously optimize alternative biofuel production processes to gradually replace traditional fossil fuels. One of the biofuels that has been largely studied is biodiesel, which is a mixture of fatty acid methyl esters obtained from triglycerides through chemical reactions with the aid of homogeneous or heterogeneous catalysts [5]. The ease of separation of the heterogeneous mixture, the possibility of reusing the catalyst, and the reduction of production costs, are certain advantages of heterogeneous catalysts over its homogeneous counterparts [6,7,8]. Currently, large-scale biodiesel production relies on the use of CaO as heterogeneous catalyst, due to its low cost and good efficiency [9,10]. However, CaO is known to be chemically unstable when exposed to ambient conditions, active sites on the surface of CaO are quickly poisoned due to the chemisorption of water and CO_2_ present in the air, triggering the formation of hydroxyl carbonates. This presents a technical disadvantage since thermal treatments to reactivate or preserve the CaO catalyst hinder the performance of the catalyst in the biodiesel production [11]. Several studies have demonstrated the feasibility of using non-conventional sources in the production of catalysts, which are subsequently employed in transesterification reactions to obtain biodiesel. This approach replaces the use of commercial reagents, decreasing production costs and wastes associated. Most common non-conventional sources employed are based on calcium-rich biowaste, such as shells from different organisms [12,13,14], and waste chalk (CaSO_4_·2H_2_O) [15]. Moreover, alternative materials with better physicochemical properties have been explored to replace CaO as heterogeneous catalysts in transesterification reactions. Among them, several materials and composites such as CaTiO_3_, CaZrO_3_, CaO–CeO_2_, CaMnO_3_, Ca_2_Fe_2_O_5_, KOH/Al_2_O_3_, sodium titanates, lithium compounds (silicates and carbonates), have been explored [8,16,17,18]. For instance, lithium silicates, inorganic materials with good thermal and chemical stability have been proposed as potential heterogeneous catalysts in biodiesel production due to its basic sites strength, high melting point (1200 °C), and good stability toward ambient conditions, this is mainly due to its low CO_2_ absorption rate at temperatures below 250 °C [19]. Furthermore, the use of non-conventional precursors for the synthesis of lithium metasilicates has been reported using diatomaceous earth and rice husk ash as silicon sources [20,21,22,23], such materials have been also tested in the production of biodiesel with promising results as compared to their commercial reagents produced counterparts. 

This study describes the development of a versatile, simple, and low-cost method to synthesize lithium metasilicate (Li_2_SiO_3_) from non-conventional silicon sources, two different sand sources from the central area of Mexico. The structural and physicochemical properties of the obtained materials were evaluated by XRD, SEM-EDS, XPS, Raman, N_2_ adsorption–desorption, and Hammet titration to select the best candidate for its application as heterogeneous catalyst in the soybean oil transesterification reaction. Remarkably, the produced lithium metasilicate materials displayed good chemical purity, with high crystallinity, and good synthetic yields (up to 89.53%). An experimental design 2^3^ was implemented considering the following parameters: methanol/oil ratios (6:1 and 18:1), catalyst weight (1 and 5%), and reaction time (1 and 3 h), for the biodiesel production. 

## 2. Materials and methods

*Materials.* LiOH 98% (Sigma Aldrich, St. Louis, MO, USA), and H_2_SO_4_ 98% (Sigma Aldrich) were used as received. Sand source 1, sand source 2, taken from different mineral deposits located in the central region of Mexico were employed as silicon sources. To facilitate the identification of the silicon sources and samples, they will be further referred to as: PE = sand from Pedro Escobedo, TEQ = sand from Tequisquiapan, LMRFPE = lithium metasilicate from PE source, LMRFTEQ = lithium metasilicate from TEQ source, and LMConv = lithium metasilicate from commercial reagents (used as a reference).

For the synthesis of biodiesel, anhydrous methanol—reactive grade (J.T. Baker, Phillipsburg, NJ, USA), and commercial soybean oil (Imperial^®^, The Calvario, Tehuacan, Puebla, México) were used. The resulted biodiesel was evaluated by GC/MS following the ASTM D6584-17 method [24]. For gas chromatography analysis, HPLC-grade pyridine (Avantor Performance Materials, Inc., Center Valley, PA, USA), n-heptane (Sigma Aldrich), methyl heptadecanoate (Sigma Aldrich), and fatty acid methyl ester standards (Sigma Aldrich) were used. N-Trimethylsilyl-N-methyl trifluoroacetamide (MSTFA) was obtained from Agilent (Agilent technologies, Santa Clara, CA, USA) and used as received. 

*Silicon sources pre-treatment*. The silicon sources were ground, mixed with NaOH, and calcined (550 °C, 2 h). Subsequently, the solid obtained was suspended in a 1 L of deionized water, sulfuric acid was then added until the suspension was neutralized (this step regulates the amount of OH^−^ ions), then the suspension was filtrated and the solid obtained dried at 80 °C for 2 h. 

**Figure 1:**
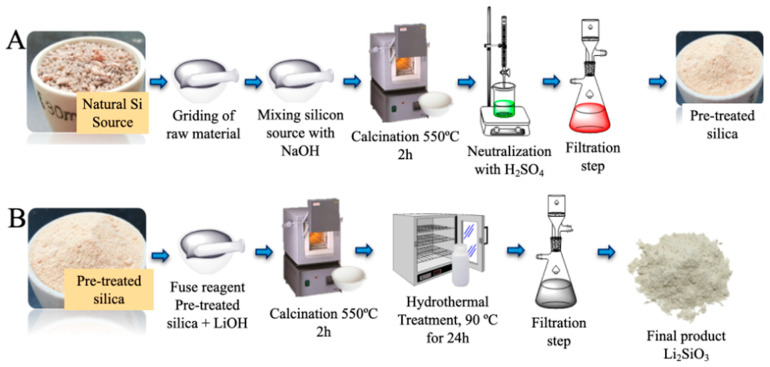
(**A**) Natural Si sources pretreatment procedure scheme; (**B**) methodology applied to produce lithium metasilicates from natural Si sources.

*Synthesis and characterization of lithium metasilicate.* For the production of lithium metasilicate (Li_2_SiO_3_), a solid-state reaction followed by a hydrothermal process was employed (Figure 1). The pretreated silicon sources were ground, sieved, and thoroughly mixed with a certain amount of LiOH in a mortar (1/1.25 wt.% ratio). Subsequently the mixture was calcined at 250 °C for 30 min, then the mixture was cooled down and homogenized in a mortar, subsequently the sample was calcined at 550 °C for 2 h (fuse reagent process). Once the fuse reagent process was completed, the obtained material was suspended in 180 mL of deionized water and placed inside a sealed polypropylene reactor for hydrothermal synthesis (90 °C for 24 h), after this period the reactor was quenched and the suspension was filtered, the recovered powder was washed several times with deionized water followed by a drying step at 90 °C for 2 h and stored in a dry place.

*Hammet titration*. Hammett titration was implemented to investigate the basic strength and chemical stability of the obtained samples, titration was also conducted on two references (CaO and LMConv). The following parameters were evaluated for the potential samples to be implemented in the biodiesel production process: (i) chemical purity of the silicon source, (ii) production efficiency of lithium metasilicate after hydrothermal process, (iii) chemical and crystalline phase purity of the obtained materials, and (iv) stability of the materials toward ambient conditions. 

*Biodiesel production.* The detailed procedure has been described elsewhere [17], the reactor was initially filled with methanol and a certain amount (1 wt.% and 5 wt.%) of catalyst. The reactor was heated for 30 min at a temperature of 60 °C under vigorous stirring. Finally, the previously filtered soybean oil was added to the three-neck flask implemented with a reflux system. After the transesterification reaction, the catalyst was removed by centrifugation, and the supernatant was heated at 50 °C for 30 min to evaporate the residual methanol obtaining the fatty acid methyl esters (FAMEs). The experimental design for the transesterification reactions considered three parameters for biodiesel production: methanol—soybean oil ratio (mol/mol) (6:1, 18:1), weight of catalyst used (1, 5 wt.%), and reaction time (60 and 180 min). 

## 3. Results and Discussion 

Non-conventional sources (PE and TEQ sand) were evaluated using XRD and EDS (see Supporting Information Figures S1 and S2). Figure S2 shows the atomic percentages (wt.%) of both non-conventional sources. PE sand possesses a high potassium and sodium concentration. Furthermore, the non-conventional TEQ sand source displays higher amounts of calcium, magnesium, and iron. These concentrations of different cations may affect the pretreatment [25] of non-conventional sources and eventually the synthesis of Li_2_SiO_3_, this will be further discussed in the following sections.

### 3.1. Synthesis of Lithium Metasilicate (Li_2_SiO_3_)

A two-step process to prepare lithium metasilicate was implemented in this study. The first stage involved the purification of the silica source, through a solid state reaction, to remove potential impurities (i.e., aluminum or earth-alkaline cations commonly present in sand sources) in the final catalyst that could hamper their performance in the biodiesel production. Subsequently, a hydrothermal treatment was employed to perform the dissolution and crystallization of the precursors into the targeted material, obtaining high reaction yield and high purity in the final products. Lithium silicates have been thoroughly used in several applications; for instance, Li_4_SiO_4_ and Li_2_SiO_3_ have been used for CO_2_ capture and as basic catalyst, respectively. The formation of Li_4_SiO_4_ by solid state is possible at temperatures of 550 °C using LiOH or LiNO_3_, nevertheless, sublimation of Li can occur at high temperatures affecting the yield in the solid-state reaction; therefore, an excess of Li precursor is needed to compensate for this loss [26]. In the present work the formation of Li_2_SiO_3_ following a less harsh experimental condition was executed for developing a sustainable synthetic route. Moreover, the use of sand as silicon source was demonstrated as well as the solid transformation of Li_4_SiO_4_ into Li_2_SiO_3_ by means of hydrothermal treatment indicating that this new route could be considered as ecofriendly as compared to conventional high-energy procedures where temperatures above 1000 °C are commonly employed. Typically, Li_2_SiO_3_ has been synthesized from hydrothermal treatments according to the following reaction (Equation (1)) [27]:

2Li^+^ + SiO_2_-H_2_ + 2OH^−^ → Li_2_SiO_3_ + 2H_2_O (1)

Basic hydrothermal system LiO_2_-SiO_2_-H_2_O is very well understood and it is clear that the Ostwald ripening occurs forming, as the latter phase, Li_2_Si_2_O_5_ depending on experimental conditions. Then, the kinetics in the synthesis is critical for obtaining a pure Li_2_SiO_3_ phase from this synthesis procedure [26]. According to Alemi and co-workers [28], formation of Li_2_SiO_3_ and Li_2_SiO_5_, by hydrothermal method, occur when there exists a Li:Si atomic ratio of 1:2 and 1:3, respectively. This information is vital to explain the Li_2_SiO_3_ formation reported herein, considering that when the Li_2_Si_2_O_5_ is formed, this phase is dissolved in the aqueous medium (by Ostwald ripening) increasing the Si concentration until obtaining a Li:Si atomic ratio of 1:2. This idea is further supported by Bennington and co-workers [29], where the enthalpies of formation of Li_2_SiO_3_ and Li_2_Si_2_O_5_ of −393.8 ± 0.8 and −611.8 ± 0.1 kcal/mol, were reported respectively. According to Gibbs energy concept, ∆*G =* ∆*H − T*∆*S*, meaning that high negative values of ∆*H* increase the ∆*G* negative values; then Li_2_Si_2_O_5_ with a high negative ∆*H* is first formed although, Ostwald ripening, this phase is transformed to Li_2_SiO_3_. Moreover, formation of non-stoichiometric compounds of lithium silicate can occur allowing the incorporation of another element such as Al which, in the case of transesterification reaction, could be detrimental.

### 3.2. Lithium Metasilicate Characterization

**Figure 2:**
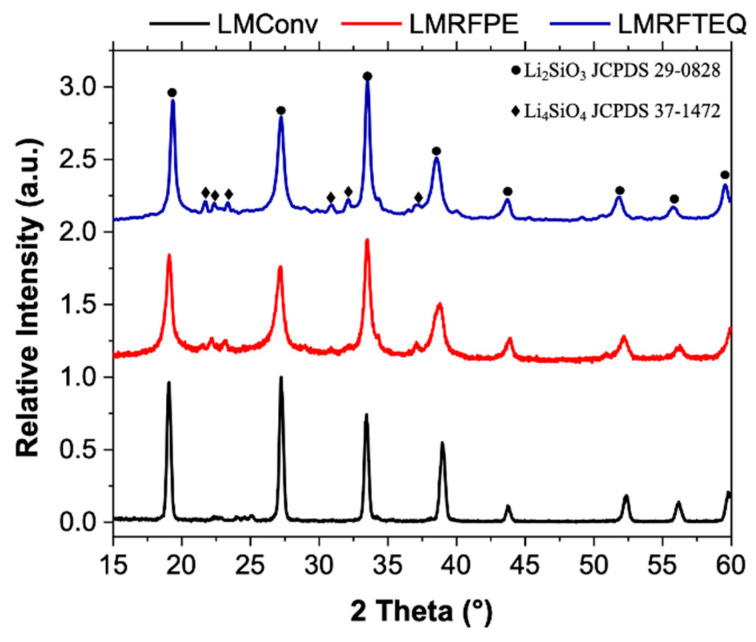
XRD diffraction patterns of lithium metasilicate synthesized using non-conventional sources, compared to LiMET synthesized from reagent grade precursors.

The XRD diffraction patterns of lithium metasilicate (LiMET) synthesized from non-conventional sources, compared to a sample of LiMET synthesized from conventional (reagent grade) sources (LMConv), are depicted in Figure 2. The three diffraction patterns show eight characteristic peaks of Li_2_SiO_3_ (JCPDS 29-0828), corresponding to the crystallographic planes (020), (111), (130), (211), (132), (241), (330) [30]. However, the diffraction patterns of LMRFPE and LMRFTEQ display weak signals corresponding to lithium orthosilicate-type material (Li_4_SiO_4_; JCPDS 37-1472), as reported by Dai and co-workers [24] at 21.6°, 22.41°, 23.38°, 30.9°, 32.14°, and 37.05° (2θ). 

**Figure 3:**
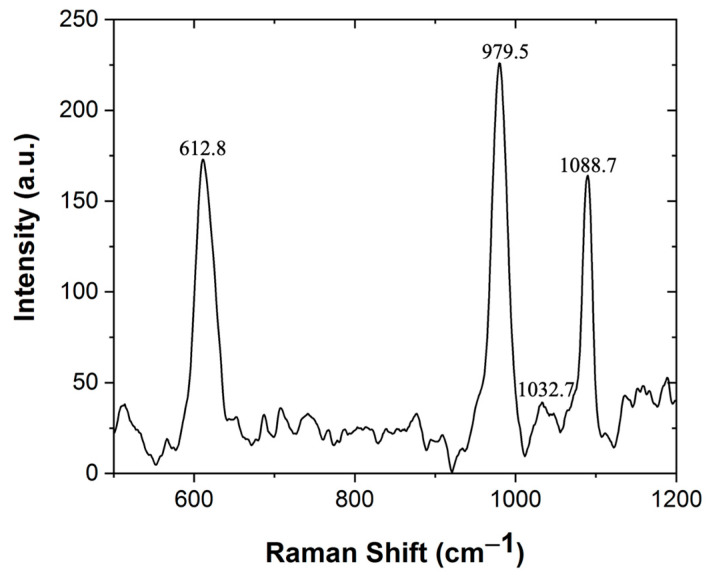
Raman spectrum of LMRFPE, corroborating the XRD results (Figure 2), where the characteristic signals of a mixture of Li_2_SiO_3_ and Li_4_SiO_4_ in small proportions was observed. Raman analysis was performed using a Renishaw model inVia with a microscope objective of 20× and the 785 nm laser.

Lai and co-workers [31] reported that the diffraction patterns of Li_2_SiO_3_ and Li_2_Si_2_O_5_ could appear at the same XRD angles (2θ). To rule out this possibility, Raman experiments were conducted (see Figure 3), characteristic Raman signals of LMRFPE were observed at 613 cm^−1^, 737 cm^−1^, 921 cm^−1^, 977 cm^−1^, and 1032.7 cm^−1^, which are in agreement with those previously reported in the literature for Li_2_SiO_3_ [32]. Additionally, only the characteristic signal (1088 cm^−1^) corresponding to the O-Si-O vibration of Li_4_SiO_4_ was spotted, demonstrating that the obtained material contains a mixture of mostly lithium metasilicate and in small quantities lithium orthosilicate, without the presence of Li_2_Si_2_O_5_ [31,32].

**Figure 4:**
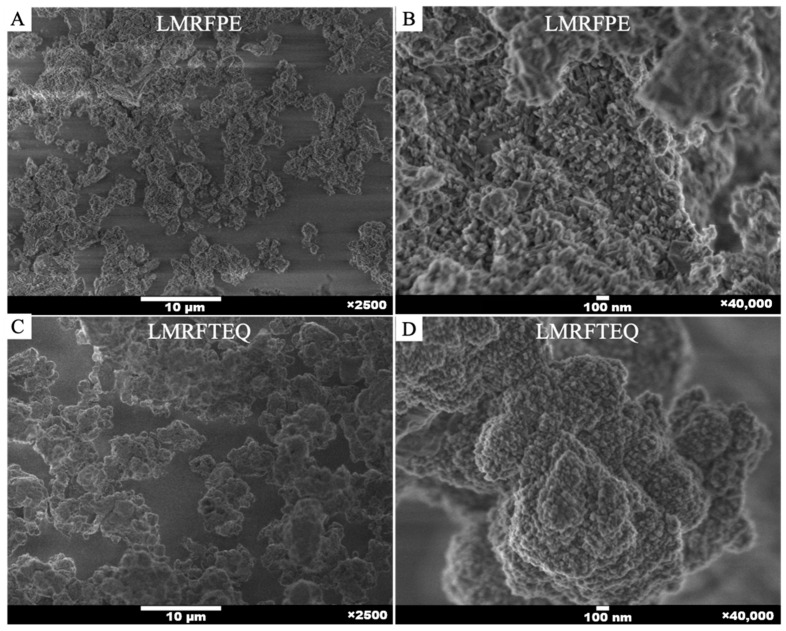
Comparison of the morphological structure of the synthesized materials. Micrographs (**A**) and (**C**) ×2500 magnification; (**B**) and (**D**) ×40,000 magnification.

The morphological analysis of the synthesized LiMET was carried out by SEM, Figure 4 shows the micrographs of LMRFPE and LMRFTEQ. In both cases, agglomerated crystals with rough and irregular surfaces were obtained. A size distribution analysis was conducted using ImageJ software, where the size of the crystals varies in the range from 1 μm to 10 μm in each of the obtained products (see Supporting Information Figure S3). 

The basic strength and chemical stability of Li_2_SiO_3_ are presented in Table 1, where the chemical resistance to air of LMRFPE and LMRFTEQ was compared against two references, LMConv and CaO (Sigma Aldrich), in which CaO displays lower stability after 24, 48, and 72 h. CaO has strong chemisorption of H_2_O and CO_2_ molecules, leading to a loss of stability when exposed to air for short periods (3 min) [19]. On the other hand, Li_2_SiO_3_ is less sensitive to CO_2_ at room temperature and is much more resistant to aging, obtaining similar results as those reported for Li_4_SiO_4_ [22] (see Table S1 support information).

Hammet titration is an indicator of basicity of catalysts; in the case of LMRFPE the basicity did not suffer a significant change, whereas, in the case of CaO, their basicity was affected due to instability under aging conditions. This result guarantees the potential reusability of the Li_2_SiO_3_ prepared using non-conventional materials in subsequent catalytic rounds. 

**Figure 5:**
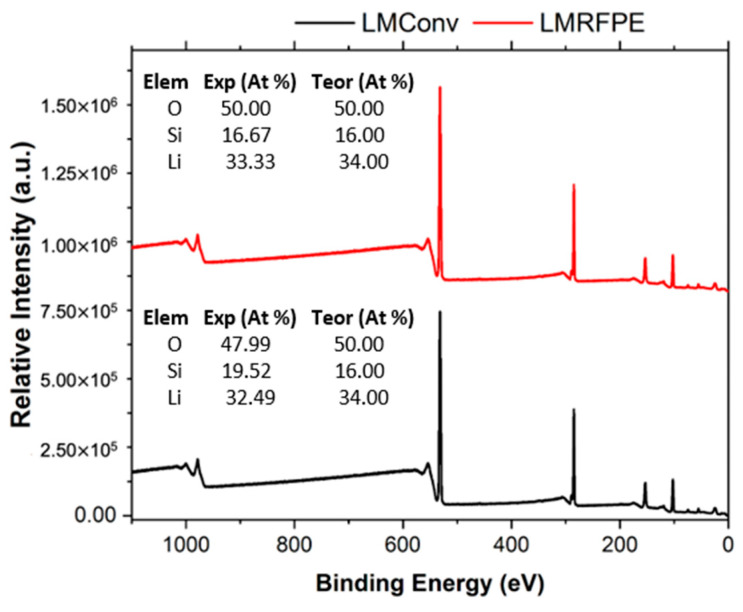
XPS elemental analysis comparison of LMRFPE (red line) and LMConv (black line), displaying a high purity of the synthesized materials. XPS analysis was performed using a K-ALFA^TM^ equipment (Thermo Fisher Scientific, Walthamm, MA, USA).

LMRFPE was selected to be employed as the catalyst for the transesterification reaction (biodiesel production) for the following reasons: (i) the sand precursor contains a higher concentration of potassium (Figure S2), which can be associated with a decrease in the material’s melting point during its pretreatment [25], facilitating the removal of cations during the Li_2_SiO_3_ synthesis. (ii) The diffraction pattern of LMRFPE (Figure 2) shows a lower amount of Li_4_SiO_4_ than that of LMRFTEQ. (iii) The chemical stability of LMRFPE is higher at 0, 24, 48, and 72h as compared to LMRFTEQ. (iv) Finally, X-ray photoelectron spectroscopy (XPS) measurements indicated a high chemical purity of LMRFPE as compared to LMConv. Figure 5 depicts a comparison of LMRFPE and LMConv elemental analysis employing XPS, showing that both materials present atomic percentages very similar to the Li_2_SiO_3_ theoretical percentages. 

**Figure 6:**
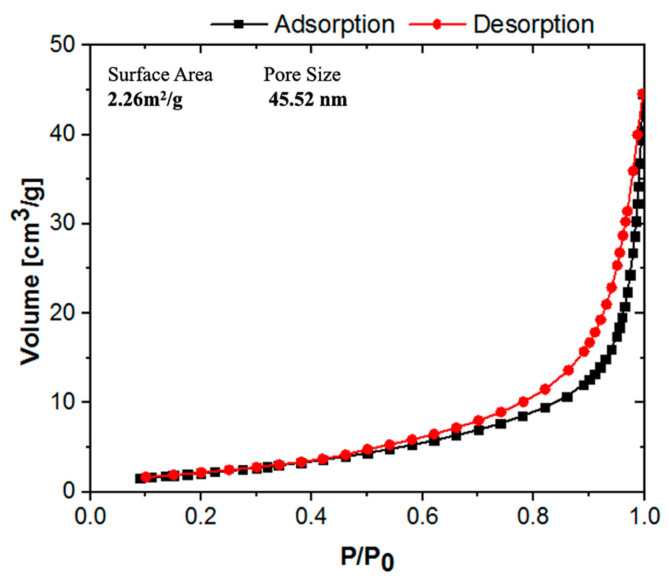
N_2_ Adsorption–desorption of LMRFPE. The isotherm displays a type IV (according to the IUPAC) showing an hysteresis loop H3. Gas adsorption measurement was conducted on a MicroActive for TriStar II Plu device, using N_2_ in the adsorption analysis at 77.35 K.

To evaluate the textural properties of LMRFPE, N_2_ adsorption–desorption experiments were conducted using the Brunauer–Emmett–Teller (BET) method, which resulted in a surface area of 2.26 m^2^/g and pore size of 45.52 nm. The isotherm obtained for LMRFPE (Figure 6) is typical of mesoporous adsorbents (type IV) with a hysteresis loop characteristic of agglomerates particles (H3) [33,34,35]. LMRFPE surface area result is in agreement with the value reported by Dai and co-workers [24], in which the Li_2_SiO_3_ synthesized by solid-state reaction and used for transesterification reaction, displayed a surface area of 1.90 m^2^/g.

### 3.3. Biodiesel Production (Transesterification Reaction)

An experimental design 2^3^ was implemented to determine the best parameters for biodiesel production using the most promising sustainable catalyst (LMRFPE), using different weight percentages (1, and 5%), subjected to different reaction times (60, and 180 min), and different molar ratios of methanol/soybean oil. Table 2 shows the results obtained for each of the parameters analyzed, where the highest yield in the production of fatty acid methyl esters (FAMEs) was 95.5%, under the following conditions: 5% catalyst, 180 min of transesterification reaction, and a molar ratio of 18:1. These results are shown in Figure S6 (Supporting Information), which displays the different parameters used in the transesterification reactions presented in Table 2.

The results obtained in the production of biodiesel are consistent with previously reported studies, where a higher yield was obtained when high weight percentages of the catalyst (5%) were employed, increasing the presence and availability of active sites, favoring the interaction between the catalyst and the reagents [36]. The reaction time used during the transesterification reaction was not longer than 180 min to avoid the possible generation of byproducts and the possibility of promoting the hydrolysis of the esters produced (saponification) [18]. Finally, an excess of methanol was used in the molar ratio with soybean oil to promote the equilibrium in forming FAMEs [19]. Statistical analysis of FAMEs yields was conducted using the Statgraphic software version 19 (Statgraphics Technologies, Inc., The Plains, VA, USA). Table 3 shows the ANOVA of the implemented experiments.

**Figure 7:**
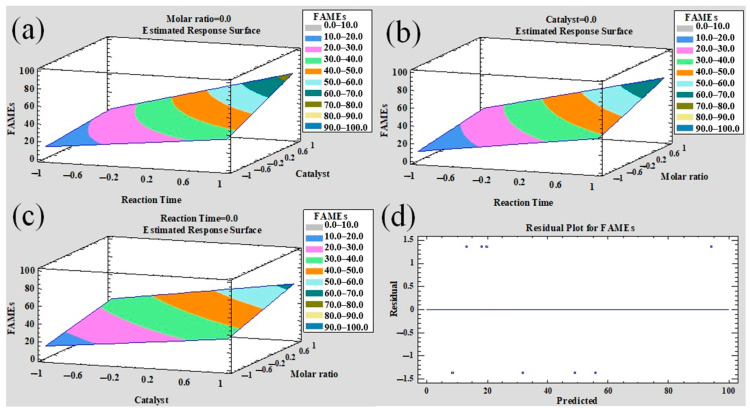
(**a**–**c**) Show the surface response of FAMEs production as a function of the studied factors. In these figures, the FAMES production increases according to the catalyst, reaction time, and methanol: oil molar ratio. Moreover, according to R^2^ = 99.74% value, as well as the lack-of-fit test (not showed), the selected linear model is appropriated to realize the optimization or the estimation of maxima value of the response. Then, the indicated contours in the figures show the path to reach the maxima value of FAMEs production with the different combinations of factors. It is possible to note that the maxima production of FAMES is achieved with extreme conditions of the studied experimental factors. (**d**) shows the residual values in function of the predicted values calculated from Equation (2). The random distribution of points is observed indicating that the model follows the assumption of normality required also to validate the statistical analysis and results.

Table of ANOVA shows the experimental factors with significant effect to FAMEs yield. Results indicate that the reaction time is the principal factor for the FAMEs production. Moreover, experimental factors have a positive effect on the response indicating that the FAMEs yield increases with the high level of factors to obtain high production of biodiesel under the following conditions: 5% catalyst, 180 min of transesterification reaction, and a methanol-oil molar ratio of 18:1, obtaining an estimated yield of 94.13%. Residual value (estimated value—real value) is 1.37 units, which means a low experimental error and acceptable for the biodiesel production. Moreover, reaction time is the factor with the most significant positive effect for obtaining biodiesel, indicating that a large reaction time could promote an increase in the biodiesel production yield. However, energetic consumption will also increase directly, hindering the ecological added value offered by the strategy presented in this report. Surface response graphs were constructed with the regression equation (Equation (2)) generated in the statistical analysis (Figure 7).

FAMEs = 36.2625 + 18.4125A + 12.4625B + 11.9625C + 7.8625AB + 4.9125AC + 2.2625BC(2)

Surface response indicates the yield tendencies of FAMEs production; the increase of experimental value factors also increases the FAMEs production yield. This tendency is in agreement with the literature as well as with the kinetic of the transesterification reaction under the catalytic effect of Li_2_SiO_3_. Cherikkallinmel and co-workers [37] achieved biodiesel production using Li_2_SiO_3_ through Box–Behnken design, where the optimized conditions to maximize the yield of biodiesel production were reported being those close to the results obtained herein. However, in that study the authors used reagent grade and conventional precursors to synthesize the employed catalysts.

## 4. Conclusions

The development of a sustainable synthetic route to produce lithium metasilicate using non-conventional sources is presented in this report, where a two-step approach involving a solid-state reaction followed by a hydrothermal treatment yielded highly pure lithium silicate materials. Physicochemical and structural characterization showed that the obtained materials is mostly composed of lithium metasilicate with small amounts of lithium orthosilicate as a secondary phase, displaying similar chemical resistance and basic strength as those observed for commercially derived counterpart materials, opening up alternatives for its use as heterogeneous catalysts in transesterification reactions to obtain biodiesel. Best performing sustainable lithium metasilicate catalysts achieved 95.5% of FAMEs production under the following conditions; 5 wt.% catalysts, 18:1 methanol/oil molar ratio, and 180 min of reaction time. The results presented herein highlight the development of functional materials based on the valorization of minerals, demonstrating the feasibility of employing sustainable materials in advanced applications. 

## Figures and Tables

**Table 1:** Comparison of basic strength of lithium metasilicate obtained from conventional and non-conventional sources and CaO.

Hammet Titration				
Air Exposure Time (h)				
	T_0_ = 0	T_1_ = 24	T_2_ = 48	T_3_ = 72
LMRFPE	13.2 < H < 15	9.9 < H < 14.7	9.0 < H < 14.7	9.5 < H < 12
LMRFTEQ	10 < H < 14.3	9.8 < H < 14.3	8.7 < H < 13.2	8.5 < H < 12
LMConv	13.8 < H < 15	9.7 < H < 14.5	8.5 < H < 15	9.5 < H < 12.5
CaO	15 < H < 18	7.3 < H < 10.1	7.0 < H < 9.9	7.0 < H < 9.8

**Table 2:** Experimental design used for the production of biodiesel using LMRFPE catalyst.

Reaction Time (min)	Catalyst (wt.%)	Molar Ratio Methanol/Soybean Oil (mol/mol)	
		6:1	18:1
60	1	7.1	19.4
	5	14.5	30.4
**180**	1	21.1	47.6
	5	54.5	**95.5**

**Table 3:** ANOVA of transesterification reaction using LMRFPE catalyst.

Source	Sum of Squares	*p*-Value
Reaction Time (A)	2712.16	0.0470
Catalyst (B)	1242.51	0.0693
Methanol-oil (C)	1144.81	0.0722
Reaction Time:Catalyst (AB)	494.551	0.1092
Reaction Time:Methanol-oil (AC)	193.061	0.1722
Catalyst:Methanol-oil (BC)	40.9513	0.3451
Error	14.8512	
Total	5842.9	
R^2^ = 99.74%		

## Data Availability

Data presented in this study are available on request from the authors.

## Supplementary Materials

The following supporting information can be downloaded at: https://www.mdpi.com/article/10.3390/ma15196753/s1. Figure S1: XRD comparison of non-conventional sources. Figure S2: Energy-dispersive X-ray spectroscopy (EDS) of non-conventional sources (A—PE sand, B—TEQ sand) used in the synthesis of Li_2_SiO_3_. Figure S3: Histogram of crystal size distribution of the synthesized lithium metasilicate aggregates. Table S1: Comparison of basic strength of lithium metasilicate synthesized in this study (LMRFPE) and lithium orthosilicate (Li_4_SiO_4_) reported elsewhere [22]. Figure S4: High resolution X-ray photoelectron spectra of each element in the catalyst structure of Li_2_SiO_3_. Depicting a comparation of LMConv and LMRFPE elemental analysis catalysts. Figure S5: Transesterification mechanism (methanol/oil) to produce fatty acid methyl esters (Biodiesel) [38]. Figure S6: Results of transesterification reaction (biodiesel production) at (A) 60 and (B) 180 minutes (reaction time) using 1% and 5 % Li_2_SiO_3_ at 6:1 and 18:1 methanol-oil molar ratios. Figure S7: FT-IR spectra shows the characteristic peaks: 612.8 cm^−1^, 733 cm^−1^ assigned to Si-O-Si stretching vibrations; 859.6 cm^−1^, 985.6 cm^−1^ are assigned to O-Si-O stretching vibrations; 1033.5 cm^−1^ assigned to Si-O stretching vibrations, and 1439 cm^−1^ is assigned to Si=O stretching vibrations [39]. FT-IR analysis was performed on a Cary 670 FT-IR coupled to Cary 620 FT-IR, Agilent Technologies.
