# Supplementary material for: Biodiesel Production Using Lithium Metasilicate Synthesized from Non-Conventional Sources
Source: Materials (Basel). 2022 Sep 29;15(19):6753. doi: 10.3390/ma15196753 (PMC9571811; doi:10.3390/ma15196753)
Supplement: Supplementary file 1 [file materials-15-06753-s001.zip › materials-1910745-supplementary.pdf]

Supplementary Information (SI)

Biodiesel production using lithium metasilicate synthesized from non-conventional sources

E. Coutino-Gonzalez,[a*] M. Ávila-Gutiérrez,[a] A. Hernández-Palomares,[b] L. I. Olvera,[c] F.J. Rodríguez-Valadez,[b] F. Espejel-Ayala[b*]

[a] Centro de Investigaciones en Óptica, A. C. Loma del Bosque 115, Colonia Lomas del Campestre, León, Guanajuato, Mexico, 37150.

[b] Centro de Investigación y Desarrollo Tecnológico en Electroquímica. Parque Tecnológico Querétaro, s/n. Pedro Escobedo, Querétaro, Mexico, 76703.

[c] Instituto de Investigaciones en Materiales, Universidad Nacional Autónoma de México, Apartado Postal 70360, CU, Coyoacán, 04510, Ciudad de Mexico, Mexico.

AUTHOR INFORMATION

fespejel@cideteq.mx, ecoutino@cio.mx

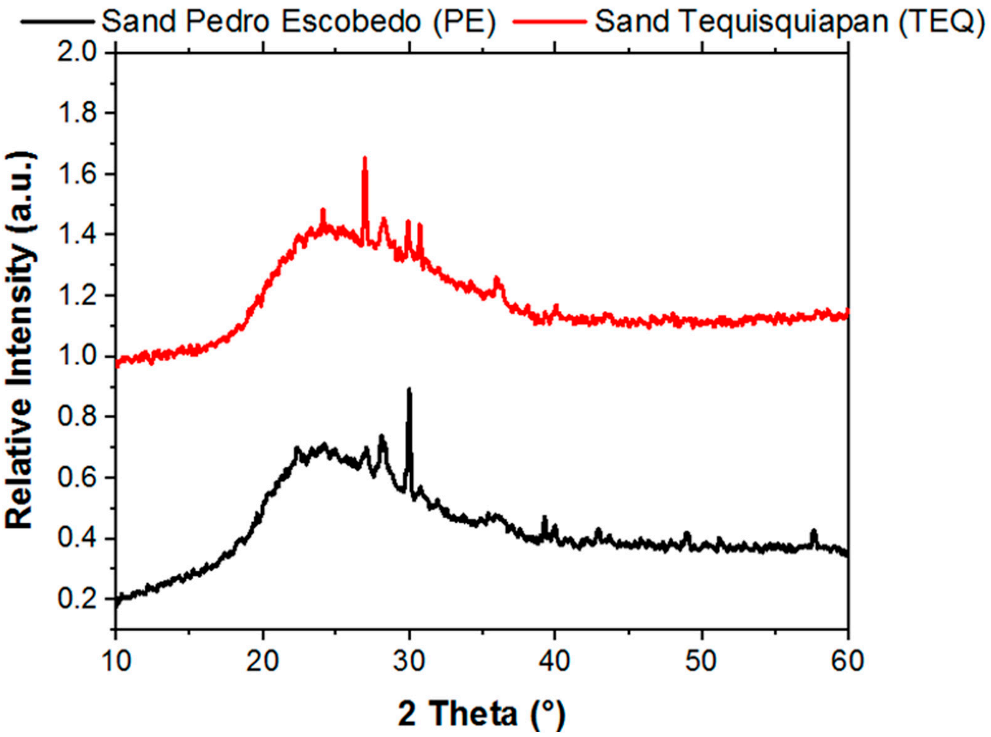

Figure S1. XRD comparison of non-conventional sources. Sand of Pedro Escobedo (black line), and sand of Tequisquiapan (red line) used as precursors for the synthesis of LMRFPE and LMRFTEQ, respectively. XRD analysis was performed with a D2 PHASER (Bruker), using Diffract.Suite software.

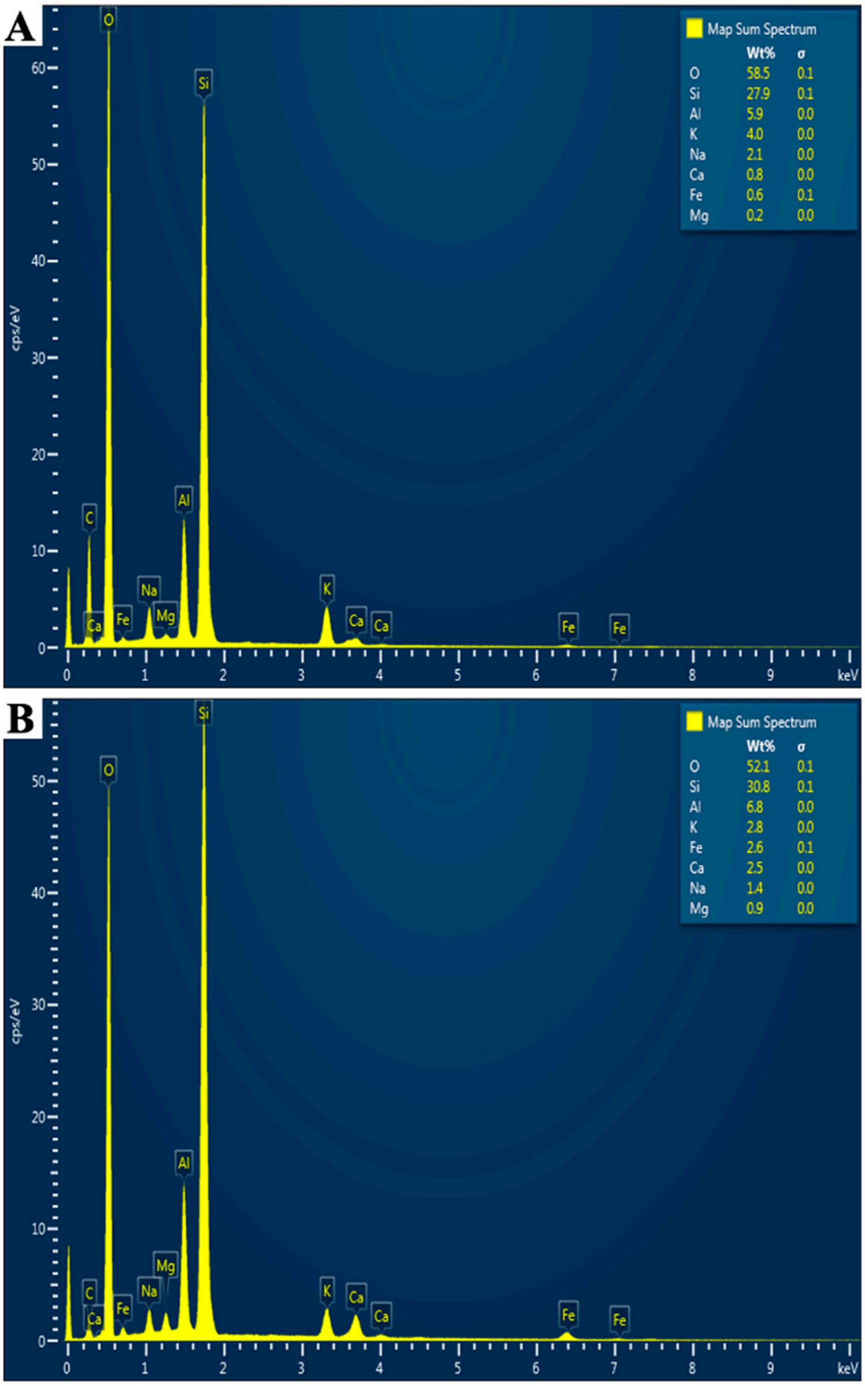

Figure S2. Energy-dispersive X-ray spectroscopy (EDS) of non-conventional sources (A – PE sand, B – TEQ sand) used in the synthesis of Li_2SiO_3. EDS analysis was performed on an Oxford Instruments X-Max coupled to a JSM-7800F Schottky Field Emission Scanning Electron Microscope.

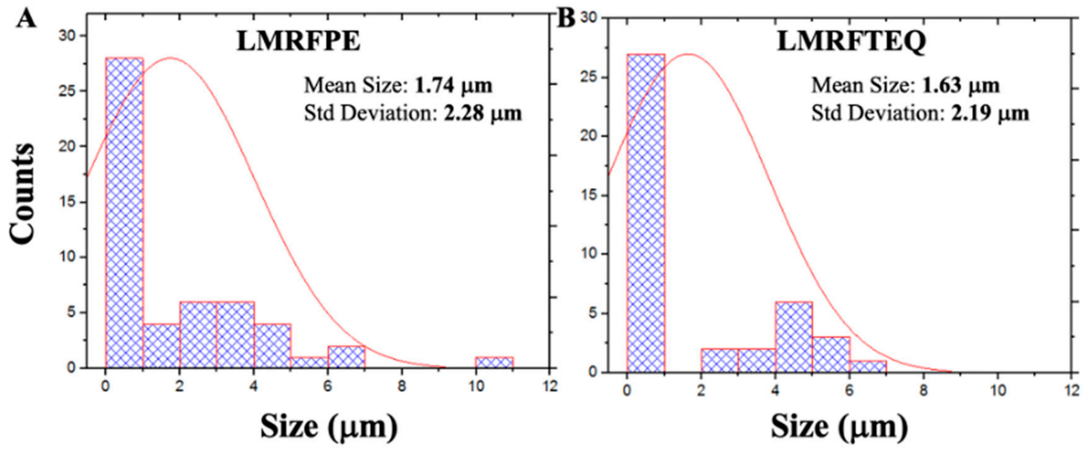

Figure S3: Histogram of crystal size distribution of the synthesized lithium metasilicate aggregates. A) LMRFPE and B) LMRFTEQ, using ImageJ software. The analysis was performed using an average of 50 crystals.

Table S1: Comparison of basic strength of lithium metasilicate synthesized in this study (LMRFPE) and lithium orthosilicate (Li_4SiO_4) reported elsewhere [1].

	**Hammet Titration**			
	**Air exposure time (hours)**			
	$T_0 = 0$	$T_1 = 24$	$T_2 = 48$	$T_3 = 72$
LMRFPE	13.2 < H < 15	9.9 < H < 14.7	9.0 < H < 14.7	9.5 < H < 12
Li_4SiO_4	12.2 < H < 15	12.2 < H < 15	12.2 < H < 15	12.2 < H < 15

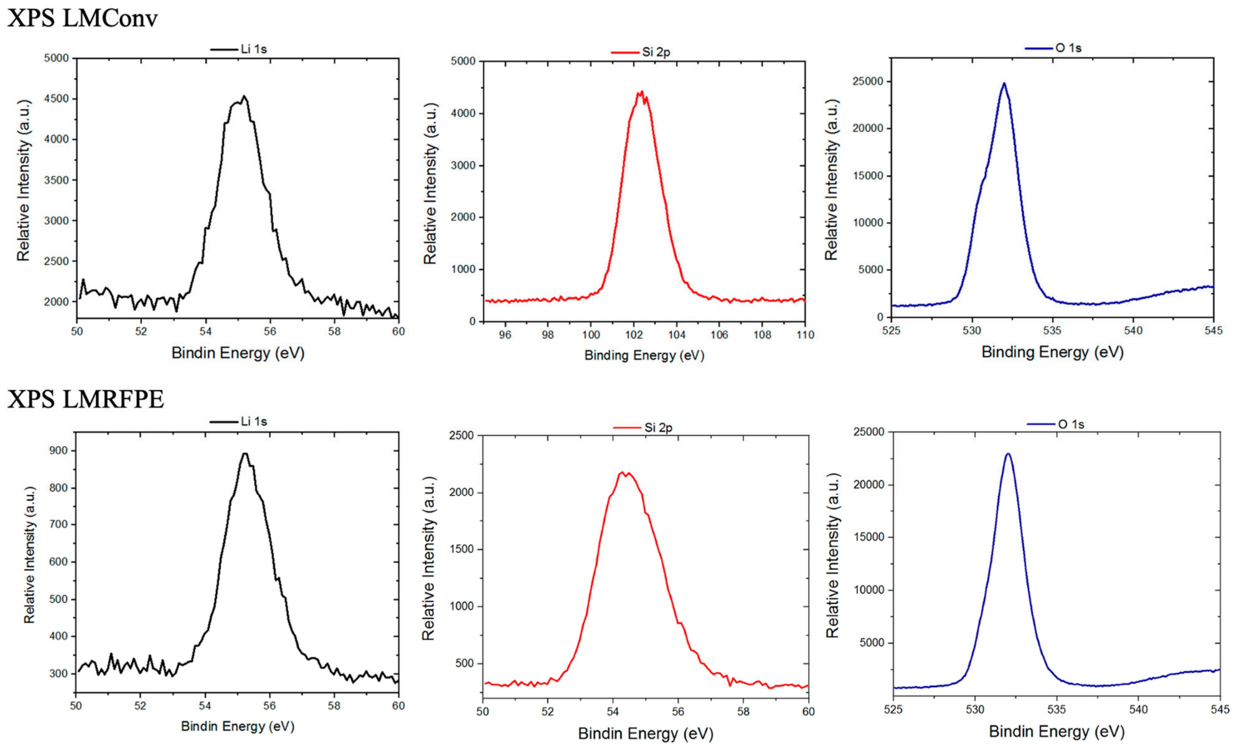

Figure S4. High resolution X-ray photoelectron spectra of each element in the catalyst structure of Li_2SiO_3. Depicting a comparation of LMConv and LMRFPE elemental analysis catalysts.

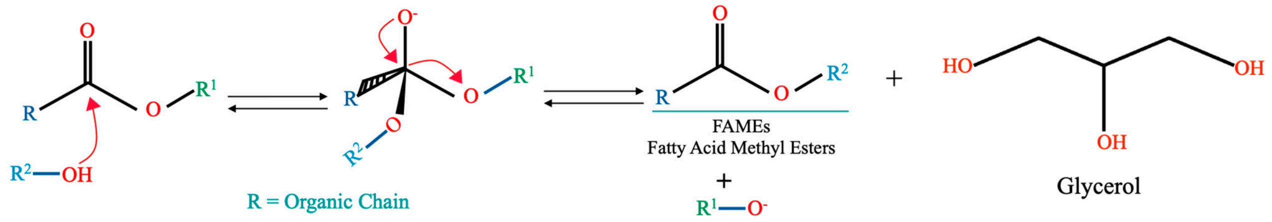

Figure S5. Transesterification mechanism (methanol/oil) to produce Fatty Acid Methyl Esters (biodiesel) [2].

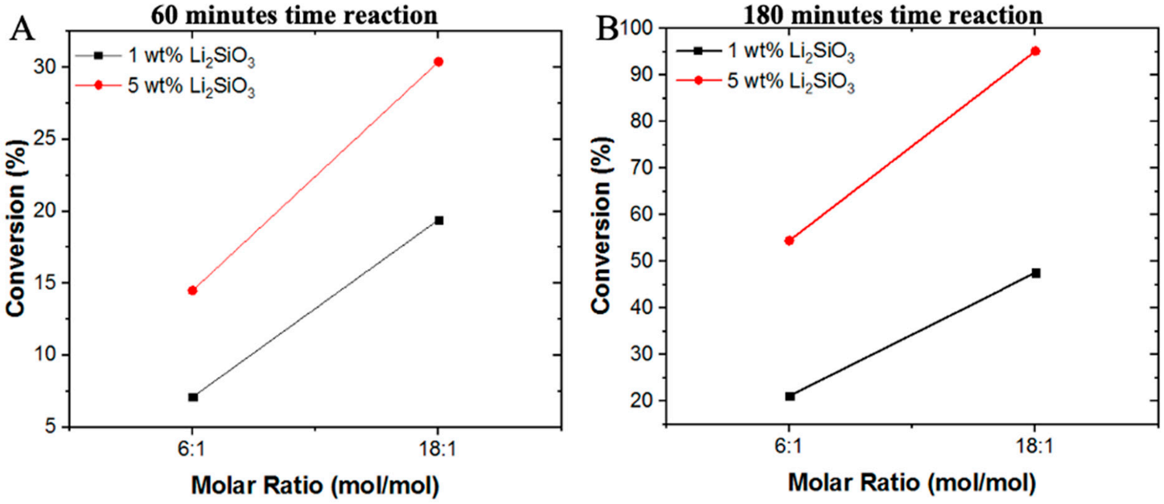

Figure S6. Results of transesterification reaction (biodiesel production) at (A) 60 and (B) 180 minutes (reaction time) using 1% and 5 % Li_2SiO_3 at 6:1 and 18:1 methanol-oil molar ratios.

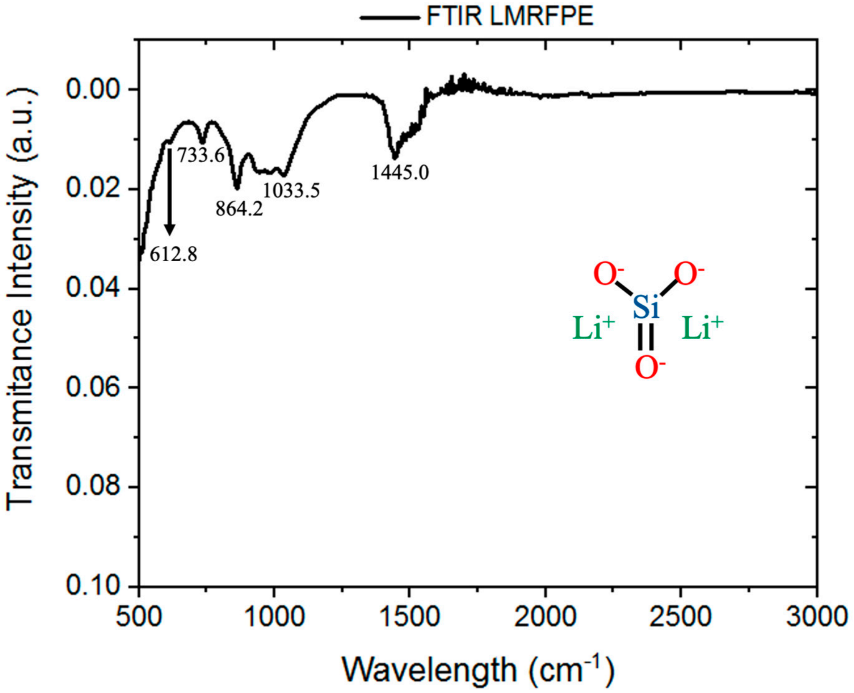

Figure S7: FT-IR spectra shows the characteristic peaks: 612.8 cm^{-1}, 733 cm^{-1} assigned to Si-O-Si stretching vibrations; 859.6 cm^{-1}, 985.6 cm^{-1} are assigned to O-Si-O stretching vibrations; 1033.5 cm^{-1} assigned to Si-O stretching vibrations, and 1439 cm^{-1} is assigned to Si=O stretching vibrations [3]. FT-IR analysis was performed on a Cary 670 FT-IR coupled to Cary 620 FT-IR, Agilent Technologies.

Reference

1. Jian-Xun Wang, Kung-Tung Chen, Jhong-Syuan Wu, Po-Hsiang Wang, Shiuh-Tsuena Huang, Chiing-Chang Chen. Production of biodiesel through transesterification of soybean oil using lithium orthosilicate solid catalyst. *Fuel Process. Technol.* **104**, 167–173 (2012).
2. Jawayria Najeeb, Sadia Akram, Muhammad Waseem Mumtaz, Muhammad Danish, Ahmad Irfan, Tooba Touqueer, Umer Rashid, Wan AzlinaWan Ab Karim Ghani, and Thomas Shean Yaw Choong. Nanobiocatalysts for biodiesel synthesis through transesterification – a review. *Catalyst* ***11**, 171, (2021).*
3. Abdolali Alemi, Shahin Khademinia. Part I: (Li2SiO3) – mild condition hydrothermal synthesis, characterization, and optical properties. *Int. Nano Lett,* **5**, 15-20 (2015).
